# CATCHing putative causative variants in consanguineous families

**DOI:** 10.1186/s12859-015-0727-5

**Published:** 2015-09-28

**Authors:** Federico Andrea Santoni, Periklis Makrythanasis, Stylianos E. Antonarakis

**Affiliations:** Department of Genetic Medicine and Development, University of Geneva, Rue Michel Servet 1, Geneva, Switzerland; University Hospitals of Geneva - HUG, Rue Gabrielle-Perret Gentil 4, Geneva, Switzerland; IGE3 Institute of Genetics and Genomics of Geneva, Geneva, Switzerland

## Abstract

**Background:**

Consanguinity is an important risk factor for autosomal recessive (AR) disorders. Extended genomic regions *identical by descent* (IBD) in the offspring of consanguineous parents give rise to recessive disorders with identical (homozygous) pathogenic variants in both alleles. However, many clinical phenotypes presenting in the offspring of consanguineous couples are still of unknown etiology. Nowadays advances in High Throughput Sequencing provide an excellent opportunity to achieve a molecular diagnosis or to identify novel candidate genes.

**Results:**

To exploit all available information from the family structure we developed CATCH, an algorithm that combines genotyped SNPs of all family members for the optimal detection of Runs Of Homozygosity (ROH) and exome sequencing data from one affected individual to identify putative causative variants in consanguineous families.

**Conclusions:**

CATCH proved to be effective in discovering known or putative new causative variants in 43 out of 50 consanguineous families. Among them, novel variants causative of familial thrombocytopenia, sclerosis bone dysplasia and the first homozygous loss-of-function mutation in *FGFR3* in human causing severe skeletal deformities, tall stature and hearing impairment were identified.

**Electronic supplementary material:**

The online version of this article (doi:10.1186/s12859-015-0727-5) contains supplementary material, which is available to authorized users.

## Background

The investigation of the molecular basis of monogenic disorders has succeeded in identifying thousands of pathogenic variants in protein-coding genes that cause these disorders. There are, however, thousands of additional (near) Mendelian phenotypes for which the molecular genetics is still unknown. Indeed, the rarity of many such disorders, the lack of statistical power due to the non-availability of large families, locus heterogeneity, and the limitations of sequencing technologies hindered the search for “Mendelian” pathogenic variants. Nevertheless, extended genomic regions *identical by descent* (IBD) in the offspring of consanguineous mattings give rise to recessive disorders with identical (homozygous) pathogenic variants in both alleles. Consanguinity is practiced in a large proportion of human populations; rates reach 20-50 % in much of the Mediterranean basin [1]. Therefore, in a consanguineous family, the search for the unknown causative gene is magnified. The typical two-step approach is to first identify extended genomic homozygous regions (ROH, Runs of Homozygosity) by genotyping all available family members with SNP arrays. Putative candidate regions are then the ROHs that are shared among all affected individuals. Second, the causative variant is finally discovered by Sanger sequencing the genes inside the candidate regions. Nowadays this slow and laborious task may be conveniently relieved by Whole Exome Sequencing (WES) of one of the affected. Indeed, it has recently been shown that combining SNP arrays and WES data is a successful approach to the identification of causative variants in homozygosity [2]. Some attempts have been made on the extraction of ROH from WES data only, but the accuracy of these methods has proven to be suboptimal with respect of the usage of SNP arrays [3]. In the future, Whole Genome Sequencing (WGS) will provide at the same time the variants with a more accurate ROH estimation than WES based approaches but, at the moment, this procedure is far from being cost-effective.

In order to integrate WES sensitivity with the optimal delineation of ROHs by SNP arrays in a comprehensive computational tool, we developed CATCH (Consanguinity Analysis Through Common Homozygosity). The algorithm recognizes affected specific ROHs from SNP array data and, inside these selected ROHs, identifies putative candidate genes from the integration of exome sequenced and annotated variants of one affected per consanguineous family.

## Implementation

### Input

CATCH takes as input: 1) the variants packaged in the standard Variant Calling Format (VCF) for one affected individual of the family; 2) a PED formatted file (http://pngu.mgh.harvard.edu/~purcell/plink/) describing the pedigree structure and the genotypes of all informative members of the family; and 3) ROH (Runs Of Homozygosity) regions as calculated by PLINK from the PED file and SNP arrays data. In this study, we used the HumanOmniExpress Bead Chip by IlluminaInc® (San Diego, CA) to genotype all family members. This SNP array tests 720 K SNPs with a mean distance of 4 kb between the SNPs. We defined as homozygous regions those regions with 50 consecutive homozygous SNPs. Exome was captured using SureSelect Human All Exons. Sequencing was performed with the Illumina HiSeq2000 and row reads were aligned with BWA [4]. Variant calling has been performed with SAMtools [5] and Pindel [6].

### Data processing

**Fig. 1:**
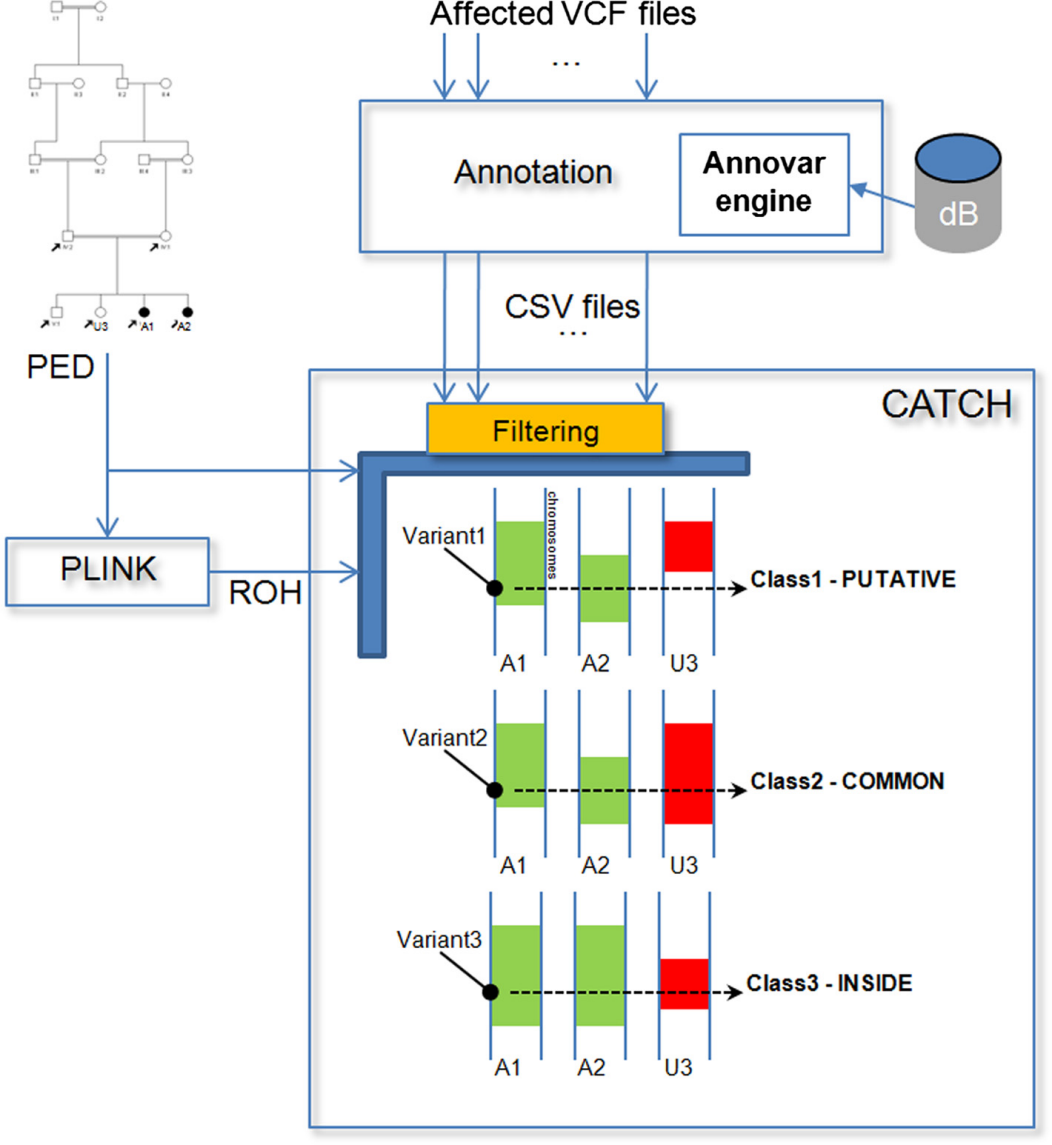
Schematic showing CATCH strategy for the identification of putative causative variants. Variants provided by a standard variant calling pipeline (i.e. BWA + Samtools or GATK ) are annotated by Annovar and filtered according to user preferences. ROH are calculated from SNPArray data by Plink for all available affected and unaffected family members. CATCH classifies every variant according to its presence/absence in ROHs as depicted in the figure. Green and red areas represent affected (A) and unaffected (U) ROH respectively

CATCH makes use of Annovar [7] to annotate sequenced variants. After, it discards non-splicing or non-exonic, synonymous, heterozygous and frequent variants in the general population (variant with MAF < 2 % in 1000 Genomes are retained [www.1000genomes.org; the results presented here have been obtained with April 2012 release]). Furthermore, CATCH does not consider variants that are in duplicated regions or exceedingly strand biased (i.e., 0 reads in one strand of the alternative allele). For each selected variant found in the genome of the sequenced (affected) individual, CATCH fetches for the related ROH and calculates the overlap with the ROHs of the other affected family members (if available) and the intersection with the respective ROH of all remaining unaffected individuals of the family. If an overlap is found, in order to exclude that the regions are identical by state (IBS), CATCH additionally considers the SNPs in the ROH surrounding the variant and evaluates the eventual concordance with the haplotypes of all family members allowing for 1 % mismatch (Fig. 1). An important exception is when the ROH of the unaffected is smaller than the overlapping ROH of the affected. In this case affected and unaffected individuals may be *identical by state* (IBS) for that haplotype block but the origin of the haplotype is actually different. In general the haplotype size depends on age, smaller being older and younger being longer [8]. Therefore, long and younger haplotypes could include a recent, deleterious variant that can be transmitted to the affected individuals along with its entire haplotype block in homozygosity through the imbreeding loops [9]. Unaffected individuals may inherit one copy of this haplotype and one copy of the older one, thus being IBS for the smaller haplotype. We found an example of such a variant in the gene *VLDLR* [10].

In summary, each variant in homozygosity is assigned to one of the following classes:

1. Class1 (Putative): neither overlap with ROH regions nor IBD has been detected with unaffected individuals.
2. Class2 (Common): IBD with some unaffected individual has been detected.
3. Class3 (Inside): ROH of the affected is longer than the overlapping ROH of the unaffected (IBS).

The output is provided as a comma separated plain text containing the annotated variants and the class they have been assigned by CATCH.

### Ethics approval

The study was approved by the Bioethics Committee of the University Hospitals of Geneva (Protocol number: CER 11–036).

## Results

As its first application, CATCH has been employed on processed samples collected from 50 consanguineous families suggestive of AR of inheritance and a wide spectrum of AR phenotypes [10]. Briefly, all samples were genotyped with a dense SNP array (HumanOmniExpress Bead Chip by Illumina) to identify Runs of Homozygosity and exome sequencing on the Illumina HiSeq2000 was performed on one affected individual per family. Prior to CATCH, raw fastq files have been processed through a custom pipeline composed by BWA [4], samtools [5] rmdup and (i) samtools mpileup for the detection of Single Nucleotide Variants (SNV) (ii) Pindel [6] for the detection of insertions and deletions. All tools were run with default parameters. On average, 21,719 variants were identified per patient. ROHs were calculated by PLINK as stretches of 50 homozygous consecutive SNPs irrespective of the total length of the genomic region, allowing for one mismatch. We considered this as a reasonable trade-off between catching a significant amount of ROH (Additional file 1: Figure S1) and limiting the number of small IBS regions that are common in all individuals. Only relatively frequent SNPs (MAF >0.3) were included in the analysis. The ROH were further defined as genomic regions demarcated by the first encountered heterozygous SNPs flanking each established homozygous region. The variants that CATCH reported as belonging to Class 1 (Putative) or Class 3 (Inside) were ranked according to the following criteria:

1) *pathogenic variants*: known pathogenic variant or variant in known pathogenic gene according to the phenotype; 2) *strong candidates variants:* variant in a gene likely involved in the pathology according to supporting literature data; 3) Variant of Unknown Significance - VUS: variant predicted to be pathogenic but in a gene not known to be related to the phenotype (Additional file 1: Figure S1). For strong candidate variants, we combined information about any known function of the gene and the gene’s family, data coming from animal models or other in vitro experiments and tissue expression. Functional validation and further investigations of the clinical relevance of these variants are still ongoing.

In 18 families, CATCH clearly identified the pathogenic variant in known disease-causing genes (*Class 1* -*DMP1*, *ARFGEF, FKTN*, *SEPSECS*, *GUCY2D*, *BBS4*, *SYNE1*, *POMGNT, MTFMT, TACO1, PYGM, PRX, TUSC3, STRA6, ALDH3A2, RNASET2, MMP2 and Class 3 - VLDLR*). Detailed information about the variants are reported in (Additional file 2: Table S1). In 5 families, strong candidates were identified in genes functionally related to the phenotype and, in a further 22 families, variants of predicted pathogenicity according to by SIFT [11], PolyPhen [12] and Mutation Taster [13] were labeled as VUS. In 5 families, no reasonable candidates or VUS were identified. All discovered variants and the predicted segregations were further validated with conventional sequencing. Eventually, CATCH suggested at least one causative variant in 36 % of families which represents a substantial improvement in the ability to diagnose recessively inherited disorders in consanguineous families [14].

In three additional studies CATCH discovered the causative variants associated to three different genetic diseases.

- A highly consanguineous family from Northern Iraq presented in several members with familial thrombocytopenia with small size platelets. CATCH identified one homozygous pathogenic variant in FYB [15], a gene encoding for a cytosolic adaptor molecule expressed by T, natural killer (NK), myeloid cells and platelets, and involved in platelet activation and controls the expression of interleukin-2. Knock-out mice were reported to show isolated thrombocytopenia.
- Two sisters from a consanguineous Lebanese family were previously reported as presenting a new atypical form of sclerosing bone dysplasia [16]. CATCH identifies a potential causative variant in the gene DMP1, a transcriptional activator of osteoblast-specific genes such as alkaline phosphatase and osteocalcin [17], already associated to Autosomal Recessive Hypophosphatemic Rickets (ARHR) [18]. The variant causes the loss of a highly conserved signal sequence of 16 amino acids resulting in a complete absence of the excretion of the protein and its retention within the cells. The diagnosis was accordingly corrected, demonstrating the importance of this approach in the delineation of the molecular basis of rare diseases especially when the clinical presentation is unclear.
- Two affected brothers born to first cousin parents originating from Egypt presented with severe skeletal deformities, tall stature and hearing impairment. CATCH identified the first homozygous loss-of-function (predicted) mutation in *FGFR3* in human [19]. This gene is one of many physiological regulators of linear bone growth and normally functions as an inhibitor, acting negatively on both proliferation and terminal differentiation of growth plate chondrocytes [20]. Before this finding, all pathogenic FGFR3 mutations in humans were associated with constitutive *FGFR3* activation by impairing endochondral bone growth.

## Conclusions

The use of whole exome sequencing in the detection of causative variants in homozygosity is really effective when associated to segregation data in a familiar context. Highly consanguineous relatives share several long Runs Of Homozygosity thus they bear a large number of potential causative variants. Of course, additional exome sequencing of non-affected relatives would dramatically reduce the number of false positives. However, the same result may be obtained at a considerably lower cost by genotyping these individuals and restricting exome sequencing to only one affected patient. CATCH is the first computational tool that process ROH, genotyping and exome sequencing data in an integrated way. It is handy and efficient, needing less than 5 min to analyze a nuclear family after annotation. It is written in Python and can run on a standard computer with a reasonable amount of RAM (>1GB). CATCH is released as Linux executable.

## Availability of the software

- **Project name: CATCH**
- **Project home page:**http://seaseq.unige.ch/~fsantoni/CATCH
- **Operating system(s):** Linux
- **Programming language:** Python
- **Other requirements:** Python 2.6 or higher
- **License:** GNU GPL.
- **Any restrictions to use by non-academics:** license needed

## Consent to publish

All patients and/or parents provided their written informed consent for the analyses performed and for the publication of the results.

## Availability of supporting data

All the variants mentioned in this study have been submitted to LOVD (http://databases.lovd.nl/whole_genome/genes).

## Additional files

Additional file 1: Figure S1. Flow chart diagram explaining the process of identification and ranking of putative candidate variants. After assignment of the pathogenicity scores according to SIFT, PolyPhen (PP) and MutationTaster (MT), CATCH classifies the variants according to ROHs (see Fig. 1 and main text). Only Class I and Class III are further labeled as: pathogenic - being a known pathogenic or a predicted pathogenic variant inside a know pathogenic gene related to the phenotype; strong candidate - predicted pathogenic variant in a gene likely involved in the pathology according to supporting literature data; Variant of Unknown significance (VUS) - predicted pathogenic variant in a gene not known to be related to the phenotype; − Benign - predicted non pathogenic variants not reported as causative in the literature. (DOCX 56 kb) Additional file 2: Table S1. Class I pathogenic variants in known disease-causing genes identified in 17 consanguinous families. (DOCX 15 kb)

## References

[1] Hamamy H, Antonarakis SE, Cavalli-Sforza LL, Temtamy S, Romeo G, Kate LP, Bennett RL, Shaw A, Megarbane A, van Duijn C (2011). Consanguineous marriages, pearls and perils: Geneva International Consanguinity Workshop Report. Genet Med.

[2] Hanson D, Murray PG, O'Sullivan J, Urquhart J, Daly S, Bhaskar SS, Biesecker LG, Skae M, Smith C, Cole T (2011). Exome sequencing identifies CCDC8 mutations in 3-M syndrome, suggesting that CCDC8 contributes in a pathway with CUL7 and OBSL1 to control human growth. Am J Hum Genet.

[3] Carr IM, Bhaskar S, O'Sullivan J, Aldahmesh MA, Shamseldin HE, Markham AF, Bonthron DT, Black G, Alkuraya FS (2013). Autozygosity mapping with exome sequence data. Hum Mutat.

[4] Li H, Durbin R (2009). Fast and accurate short read alignment with Burrows-Wheeler transform. Bioinformatics.

[5] Li H, Handsaker B, Wysoker A, Fennell T, Ruan J, Homer N, Marth G, Abecasis G, Durbin R (2009). The Sequence Alignment/Map format and SAMtools. Bioinformatics.

[6] Ye K, Schulz MH, Long Q, Apweiler R, Ning Z (2009). Pindel: a pattern growth approach to detect break points of large deletions and medium sized insertions from paired-end short reads. Bioinformatics.

[7] Wang K, Li M, Hakonarson H (2010). ANNOVAR: functional annotation of genetic variants from high-throughput sequencing data. Nucleic Acids Res.

[8] Greenwood TA, Rana BK, Schork NJ (2004). Human haplotype block sizes are negatively correlated with recombination rates. Genome Res.

[9] Szpiech ZA, Xu J, Pemberton TJ, Peng W, Zollner S, Rosenberg NA, Li JZ (2013). Long runs of homozygosity are enriched for deleterious variation. Am J Hum Genet.

[10] Makrythanasis P, Nelis M, Santoni FA, Guipponi M, Vannier A, Bena F, et al.. Diagnostic Exome Sequencing to Elucidate the Genetic Basis of Likely Recessive Disorders in Consanguineous Families. Hum Mutat. 2014;35(10):1203–10. 10.1002/humu.22617 25044680

[11] Kumar P, Henikoff S, Ng PC (2009). Predicting the effects of coding non-synonymous variants on protein function using the SIFT algorithm. Nat Protoc.

[12] Adzhubei IA, Schmidt S, Peshkin L, Ramensky VE, Gerasimova A, Bork P, Kondrashov AS, Sunyaev SR (2010). A method and server for predicting damaging missense mutations. Nat Methods.

[13] Schwarz JM, Rodelsperger C, Schuelke M, Seelow D (2010). MutationTaster evaluates disease-causing potential of sequence alterations. Nat Methods.

[14] Yang Y, Muzny DM, Reid JG, Bainbridge MN, Willis A, Ward PA, Braxton A, Beuten J, Xia F, Niu Z (2013). Clinical whole-exome sequencing for the diagnosis of mendelian disorders. N Engl J Med.

[15] Hamamy H, Makrythanasis P, Al-Allawi N, Muhsin AA, Antonarakis SE (2014). Recessive thrombocytopenia likely due to a homozygous pathogenic variant in the FYB gene: case report. BMC Med Genet..

[16] Chouery E, Pangrazio A, Frattini A, Villa A, Van Wesenbeeck L, Piters E, Van Hul W, Coxon FP, Schouten T, Helfrich M (2010). A new familial sclerosing bone dysplasia. J Bone Miner Res Off J Am Soc Bone Miner Res..

[17] Gannage-Yared MH, Makrythanasis P, Chouery E, Sobacchi C, Mehawej C, Santoni FA, Guipponi M, Antonarakis SE, Hamamy H, Megarbane A (2014). Exome sequencing reveals a mutation in DMP1 in a family with familial sclerosing bone dysplasia. Bone.

[18] Feng JQ, Ward LM, Liu S, Lu Y, Xie Y, Yuan B, Yu X, Rauch F, Davis SI, Zhang S (2006). Loss of DMP1 causes rickets and osteomalacia and identifies a role for osteocytes in mineral metabolism. Nat Genet.

[19] Makrythanasis P, Temtamy S, Aglan MS, Otaify GA, Hamamy H, Antonarakis SE (2014). A novel homozygous mutation in FGFR3 causes tall stature, severe lateral tibial deviation, scoliosis, hearing impairment, camptodactyly, and arachnodactyly. Hum Mutat.

[20] Deng C, Wynshaw-Boris A, Zhou F, Kuo A, Leder P (1996). Fibroblast growth factor receptor 3 is a negative regulator of bone growth. Cell.

