# Supplementary figures and images for: CATCHing putative causative variants in consanguineous families
Source: BMC Bioinformatics. 2015 Sep 28;16:310. doi: 10.1186/s12859-015-0727-5 (PMC4587650; doi:10.1186/s12859-015-0727-5)

Supplementary Figure 1


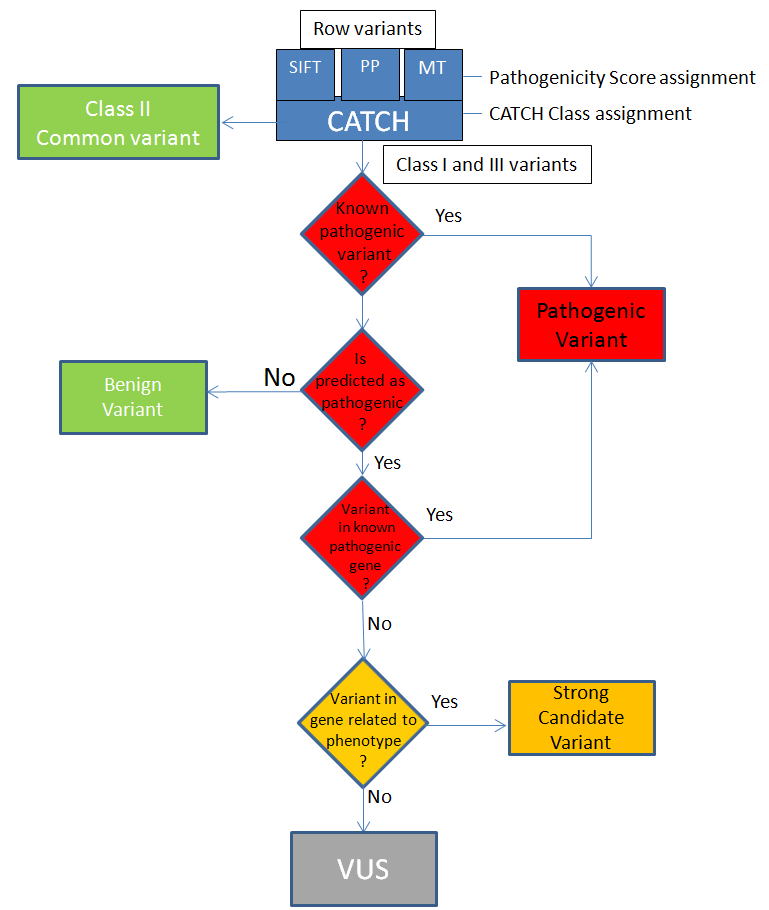

Supplement: Additional file 1: Figure S1. — Flow chart diagram explaining the process of identification and ranking of putative candidate variants. After assignment of the pathogenicity scores according to SIFT, PolyPhen (PP) and MutationTaster (MT), CATCH classifies the variants according to ROHs (see Fig. 1 and main text). Only Class I and Class III are further labeled as: pathogenic - being a known pathogenic or a predicted pathogenic variant inside a know pathogenic gene related to the phenotype; strong candidate - predicted pathogenic variant in a gene likely involved in the pathology according to supporting literature data; Variant of Unknown significance (VUS) - predicted pathogenic variant in a gene not known to be related to the phenotype; − Benign - predicted non pathogenic variants not reported as causative in the literature. (DOCX 56 kb) [file 12859_2015_727_MOESM1_ESM.docx]
