# Supplementary material for: CATCHing putative causative variants in consanguineous families
Source: BMC Bioinformatics. 2015 Sep 28;16:310. doi: 10.1186/s12859-015-0727-5 (PMC4587650; doi:10.1186/s12859-015-0727-5)
Supplement: Additional file 2: Table S1. — Class I pathogenic variants in known disease-causing genes identified in 17 consanguinous families. (DOCX 15 kb) [file 12859_2015_727_MOESM2_ESM.docx]

Supplementary Table 1

| Family  ID | Gene  (OMIM #) | Variant | dbSNP,  1000Genome freq | SIFT* | Polyphen* | Mutation  Taster* | GERP++* |
| --- | --- | --- | --- | --- | --- | --- | --- |
| Fam_1 | *DMP1*  (600980) | NM_004407:c.1A>G:  p.(Met1Val) | rs104893834,  NA | 0 | 0.61 | 0.99 | 4.3 |
| Fam_12 | *ARFGEF2*  (605371) | NM_006420.2:c.2776C>T:  p.(Arg926*) | NA | -- | -- | -- | -- |
| Fam_13 | *FKTN*  (607440) | NM_006731:c.218T>C:  p.(Phe73Ser) | NA | 0.01 | 0.86 | 0.99 | 4.01 |
| Fam_26 | *SEPSECS* (613811) | NM_016955:c.1466A>T:  p.(Asp489Val) | rs145703544,  0.00022 | 0.01 | 0.15 | 0.99 | 3.8 |
| Fam_29 | *GUCY2D*  (600179) | NM_000180:c.2563C>T:  p.(Gln855Stop) | NA | -- | -- | -- | -- |
| Fam_30 | *BBS4*  (600374) | NM_033028:c.157-3C>G | NA | -- | -- | -- | -- |
| Fam_31 | *SYNE1*  (608441) | NM_033071:c.25597dup:  p.(Ser8533Phefs*2) | NA | -- | -- | -- | -- |
| Fam_32 | *POMGNT1* (606822) | NM_017739.3:c.1539+1G>A | rs138642840,  0.000879 | -- | -- | -- | -- |
| Fam_36 | *MTFMT*  (611766) | NM_139242.3:c.17G>C:  p.(Arg6Pro) | NA | 0.02 | 0.93 | 0 | 3.91 |
|  | MAN1B1 (604346) | NM_016219.4:c.1990del: p.(Thr664Argfs*64) | NA | -- | -- | -- | -- |
| Fam_37 | *TACO1*  (612958) | NM_016360.3:c.421C>T: p.(Arg141*) | NA | -- | -- | -- | -- |
| Fam_38 | *PYGM*  (608455) | NM_005609.2:c.2447G>A: p.(Arg816His) | rs139230055,  0.000439 | 0 | 1 | 0.99 | 4.04 |
| Fam_39 | *PRX*  (605725) | NM_181882.2:c.3099del: p.(Glu1034Argfs*5) | rs139230055,  0.000439 | -- | -- | -- | -- |
| Fam_43 | *TUSC3*  (601385) | NM_006765.3:c.544A>T: p.(Ile182Phe) | NA | 0.001 | 0.967 | 0.943 | 5.17 |
| Fam_44 | *STRA6*  (610745) | NM_022369.3:c.1931C>T: p.(Thr644Met) | rs118203960,  0.00022 | 0 | 0.99 | 0.92 | 4..4 |
| Fam_46 | *ALDH3A2* (609523) | NM_000382.2:c.628G>A: p.(Gly210Arg) | NA | 0 | 1 | 1 | 5.38 |
| Fam_48 | *RNASET2* (612944) | NM_003730.4:c.115dup: p.(Met39Asnfs*7) | NA | -- | -- | -- | -- |
| Fam_49 | *MMP2*  (120360) | NM_004530.4:c.538G>A: p.(Asp180Asn) | NA | 0 | 0.99 | 1 | 4.42 |

*No score is provided for frameshift, nonsense and splicing variants.
